# Three-dimensional reconstruction and phenotype measurement of maize seedlings based on multi-view image sequences

**DOI:** 10.3389/fpls.2022.974339

**Published:** 2022-09-02

**Authors:** Yuchao Li, Jingyan Liu, Bo Zhang, Yonggang Wang, Jingfa Yao, Xuejing Zhang, Baojiang Fan, Xudong Li, Yan Hai, Xiaofei Fan

**Affiliations:** ^1^State Key Laboratory of North China Crop Improvement and Regulation, Baoding, China; ^2^College of Mechanical and Electrical Engineering, Hebei Agricultural University, Baoding, China; ^3^Hebei Runtian Water-Saving Equipment Co., Ltd., Shijiazhuang, China

**Keywords:** three-dimensional point cloud, multi-view reconstruction, maize seedlings phenotype, point cloud pre-processing, point cloud segmentation

## Abstract

As an important method for crop phenotype quantification, three-dimensional (3D) reconstruction is of critical importance for exploring the phenotypic characteristics of crops. In this study, maize seedlings were subjected to 3D reconstruction based on the imaging technology, and their phenotypic characters were analyzed. In the first stage, a multi-view image sequence was acquired *via* an RGB camera and video frame extraction method, followed by 3D reconstruction of maize based on structure from motion algorithm. Next, the original point cloud data of maize were preprocessed through Euclidean clustering algorithm, color filtering algorithm and point cloud voxel filtering algorithm to obtain a point cloud model of maize. In the second stage, the phenotypic parameters in the development process of maize seedlings were analyzed, and the maize plant height, leaf length, relative leaf area and leaf width measured through point cloud were compared with the corresponding manually measured values, and the two were highly correlated, with the coefficient of determination (*R*^2^) of 0.991, 0.989, 0.926 and 0.963, respectively. In addition, the errors generated between the two were also analyzed, and results reflected that the proposed method was capable of rapid, accurate and nondestructive extraction. In the third stage, maize stem leaves were segmented and identified through the region growing segmentation algorithm, and the expected segmentation effect was achieved. In general, the proposed method could accurately construct the 3D morphology of maize plants, segment maize leaves, and nondestructively and accurately extract the phenotypic parameters of maize plants, thus providing a data support for the research on maize phenotypes.

## Introduction

Maize, as an important food crop and one of the three major cereal crops in the world, has a high economic value (Nuss and Tanumihardjo, 2010). Maize plant height and leaf area reflect, to some extent, the plant’s growth rate and robustness, and the leaves have a significant impact on maize yield and disease resistance (Liu et al., 2021b). Therefore, accurate acquisition of maize phenotypic traits is of great significance for understanding the growth status of the crop, crop yield estimation, disease resistance detection and breeding (Fourcaud et al., 2008). Currently, phenotypic information collection of maize seedlings is an important task in the maize breeding research. Traditional research methods mainly rely on manual measurement, with the problems of high workload and low efficiency. In addition, manually collected crop phenotypes have the disadvantage of insufficient information, which greatly affects the breeding process and hinders the long-term development of breeding trials. Therefore, it is urgent to develop advanced phenotypic data acquisition techniques in modern breeding and cultivation trials (Yang et al., 2020). It is noteworthy that the high-throughput phenotyping techniques offer the possibility of rapid and nondestructive detection of crop phenotypes (Qinghua, 2018; Zhao et al., 2019). Among them, three-dimensional (3D) reconstruction techniques is more widely used in agriculture as a highly representative high-throughput phenotyping technique (Perez-Sanz et al., 2017).

At present, the application of 3D reconstruction technology in agriculture develops rapidly, with diverse methods of data acquisition (Zheng et al., 2020). For example, Moreno et al. (2020) used LiDAR system equipment for 3D reconstruction of vineyards, estimated the amount of vine pruning, and achieved considerable results. Zhou et al. (2020) analyzed the variation of vertical structure of maize plants at different inversion levels and evaluated the variation of maize plant height at different inversion levels by using an unmanned aircraft with LiDAR on board for 3D reconstruction of maize. Yang et al. (2019) proposed a 3D point cloud reconstruction method based on Kinect self-labeling with comprehensive display of fruit tree morphological information and high accuracy of parameter extraction, which could accurately extract 3D information of fruit tree canopy. Wang et al. (2020) used Kinect device for 3D reconstruction of leaf lettuce, which has high alignment accuracy and stability. Although the above methods can be used to analyze crop phenotypes, LiDAR is expensive equipment and susceptibility to weather, and Kinect device has the disadvantage of obtaining point clouds with low resolution, and the accuracy of the generated point clouds is susceptible to light.

The structure from motion (SfM) method is a 3D reconstruction technique based on the basic principles of multi-view geometry (Iglhaut et al., 2019). Generally speaking, SfM performs 3D reconstruction from the acquired multi-view images, which has the advantages of being simple to use and subject to few environmental constraints (Liu et al., 2021c). In terms of the plant phenotypic measurements, the 3D reconstruction of plants based on the SfM algorithm has some advantages of higher reconstruction accuracy as well as the ability to achieve dynamic and lossless reconstruction of the research object (Xiao et al., 2020) used the SfM method to reconstruct a 3D model of three growth stages of sugar beet in the field and extracted phenotypic traits such as height, leaf area, and leaf length. The coefficient of determination *R*^2^ > 0.8 between the measured and estimated values showed that they had a high correlation. After acquiring image sequences from three different angles, (Andújar et al., 2018) used the SfM method for 3D modeling of weed plants, and found that the actual values of plant height and leaf area could be estimated accurately. Zhang et al. (2022b) conducted a 3D reconstruction of planted forests based on the SfM method after image acquisition by UAV. The results showed that the method not only described the understory structure of the plantation forest and its centimeter-level vegetation efficiently and economically, but also constructed a large-scale point cloud model. Sun et al. (2019) reconstructed the 3D structure of cotton boll using the SfM method and got the number and location of the boll by point cloud clustering and segmentation. Additionally, Zermas et al. (2020) adopted the SfM method to reconstruct the point cloud of maize plants and obtained phenotypic parameters through extraction of their skeleton.

This paper used two methods, RGB camera photography and video frame extraction, to obtain multi-view images and then reconstructed maize seedlings in 3D based on SfM algorithm. The accuracy and speed are balanced in performing maize phenotype analysis and segmentation. In addition, a point cloud pre-processing algorithm based on Euclidean clustering algorithm, color filtering algorithm, and voxel filtering algorithm was designed, which had a good effect in obtaining maize point cloud models. Moreover, phenotypic parameters such as maize plant height, stalk height, leaf length, leaf width and relative leaf area were extracted. The dynamic changes of morphological characteristics at the seedling stage of maize were analyzed, and the accuracy of the reconstruction was evaluated based on the measured data and errors analysis. Finally, the stalk segmentation of maize seedlings was identified by using a region growth segmentation algorithm, which achieved the expected segmentation results. In summary, this study provides a convenient, rapid and quantitative analytical method for 3D phenotypic measurements of maize seedlings.

## Materials and methods

### Experimental material

The selected experimental material was the maize seed of Zhengdan 958 variety, which was purchased from Baoding agricultural market. Firstly, maize seeds were heated in a water bath at 39°C and soaked for 7 h. Then, they were planted in pots and numbered into seed germination incubator. Furthermore, the corn was observed to grow over 1 week. The temperature in the germination incubator was set to 28°C and the humidity was set to 70%. Phenotypic parameters, such as plant height, stem height, leaf length, leaf width, and relative leaf area of maize seedlings, were recorded by manual measurements. Specifically, for maize seedling height, a tape measure was adopted to measure the distance from the above-ground part of the plant to the top of the plant canopy. For leaf length and leaf width of maize, vernier calipers with an accuracy of 0.01 mm were used for measurement. The relative leaf area is approximated by the product of the measured leaf length and leaf width.

### Image acquisition

We adopted two methods to acquire images in this study. The main purpose is to balance the speed and accuracy of the corn 3D reconstruction. When analyzing the phenotypic parameters of maize, there is a higher requirement for the clarity of the point cloud model, and higher resolution images are needed. When identifying the structure of maize, it is only required to reconstruct the point cloud model quickly, and the reconstruction accuracy of the point cloud is not required at this time. In terms of the first method, the RGB camera was used to take pictures to obtain multi-view images. It required a collection of 50–60 images when the modeling was much sharper. The accuracy is also higher when analyzing phenotypic parameters of maize seedlings, but the process of data acquisition is time-consuming. The RGB camera (Model NO. FSFE-3200D-10GE, JAI) was a 2-CMOS multi-spectral prism camera. It employed two prism-mounted 3.2 megapixel CMOS imagers which were aligned with a common optical path for image alignment regardless of motion or viewing angle. The plant was placed on the center of the carrier table, and then the rotary arm with the camera rotates around it while taking photos at 6° intervals and transferring the acquired images to the computer for processing after rotating 360°.

In terms of the second method, plant videos were acquired with cellphone camera, and key frames were extracted from the video (Ma et al., 2015), with which the plant 3D model was reconstructed. The resolution of the images was 1080 × 1920, and the video frame rate was 30 fps. Modeling by video frame extraction is mainly for maize stalk segmentation recognition, which was only used to model the entire plant and has lower requirements for accuracy.

**Figure 1:**
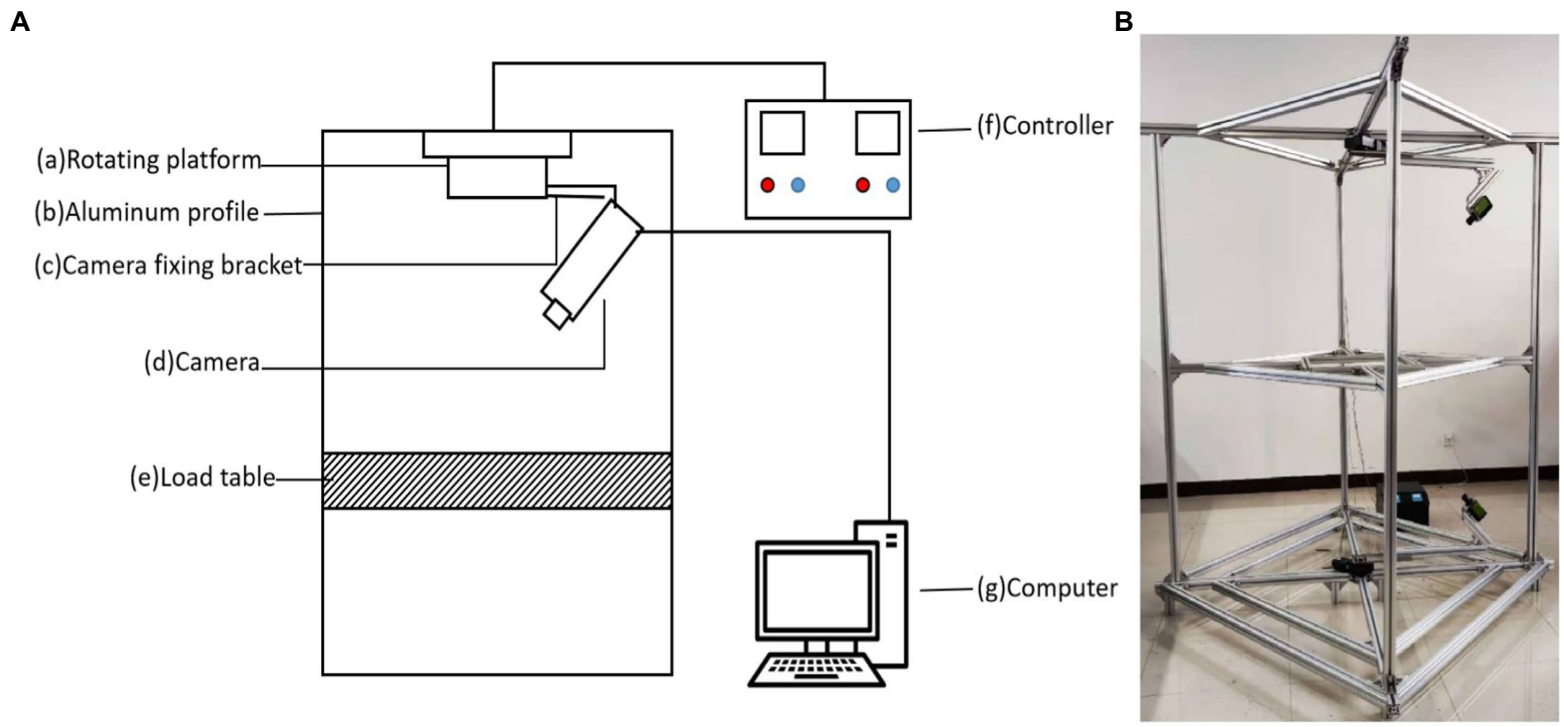
Image acquisition equipment. **(A)** Schematic diagram of the 3D imaging system. It is composed of (a) rotating platform, (b) aluminum profile, (c) camera fixing bracket, (d) camera, (e) load table, (f) controller, and (g) computer. **(B)** A picture of the 3D imaging equipment.

Figure 1A is a schematic diagram of the 3D imaging device, which consists of (a) rotating platform, (b) aluminum profile (the bracket constituting the device), (c) camera fixation support, (d) camera, (e) loading platform (for the placement of to-be-measured experimental materials), (f) controller (to control the rotational speed of the motor) and (g) computer (to process image data and for 3D reconstruction). Figure 1B is a real picture for the image acquisition part of the 3D imaging device. Therein, the rotating platform drives the rotation of the camera fixation support and camera.

### Methods

#### 3D reconstruction based on SfM

3D reconstruction techniques based on images are mainly the techniques for recovering 2D images into 3D models (Aharchi and Ait Kbir, 2019). SfM is one of the 3D reconstruction methods, and its principle is to apply the matching algorithm to the acquired multi-view image sequence so as to obtain the correspondence of the same pixel points of the image, and use the matching constraint relationship combined with the triangulation principle to obtain the 3D coordinates of the spatial points, and then reconstruct the 3D model of the object (Chen et al., 2020). The reconstruction process mainly consists of the key steps such as feature point extraction and matching, sparse point cloud reconstruction, and dense point cloud reconstruction.

In this paper, the software used for 3D reconstruction based on SfM is mainly Visual SfM (version 5.26), Agisoft Metashape (version 1.6, Agisoft LLC, St. Petersburg, Russia), and CloudCompare (Martinez-Guanter et al., 2019). For multi-view images acquired by RGB camera shooting, Agisoft metashape was used for sparse reconstruction and dense reconstruction of point clouds. For the multi-view images acquired by video frame extraction, VisualSfM was adopted to acquire the sparse point cloud, which was then reconstructed into a dense point cloud. The RGB camera used in this study, an industrial-grade camera, could acquire high-quality pictures with a big file data size, while Agisoft Metashape could process the high-quality image sequence with higher accuracy and a better effect when applied to the 3D reconstruction. The video frame extraction method, which was based on videos shot by smartphones, the resolution and image quality of the obtained image are much lower than the former, and the generated data is small. VisualSfM harvested a higher speed and smaller time consumption when used for 3D reconstruction since it supported the acceleration of GPU and CPU.

#### Point cloud preprocessing

During the acquisition of point cloud data, due to the influence of equipment accuracy and environmental factors, some noisy points may inevitably appear in the point cloud data. In addition, there are often some discrete points in the point cloud data that are far away from the subject point cloud owing to the impact of external interference factors such as line of sight occlusion and obstacles. Thus, a point cloud filtering method is needed to filter out and remove the irrelevant information, which in turn improves the speed of the point cloud processing at the time of operation (Han et al., 2017). In this paper, the Euclidean clustering algorithm was used for the removal of background (Sun et al., 2020). The color threshold-based segmentation method was utilized to remove the noise points from the plant edges (Zhang et al., 2011). The point cloud voxel filtering algorithm was then used to downsample the point cloud to reduce the number of point clouds (Miknis et al., 2016).

**Figure 2:**
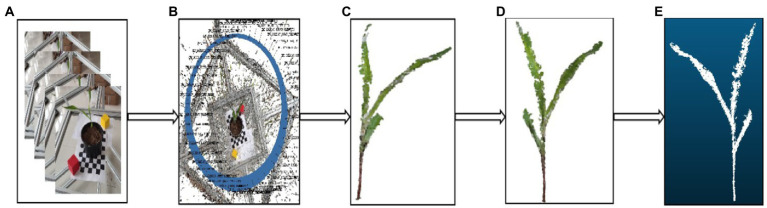
Point cloud algorithm process flow chart.

The point cloud preprocessing algorithm used in this study is as shown in the Figure 2, in which (a) displays the acquired multi-view image sequence, (b) exhibits the original point cloud data of maize acquired based on SfM algorithm, (c) is a noisy point-containing point cloud model of maize acquired through background segmentation and removal using the Euclidean clustering algorithm, (d) shows the noisy point-free point clouds of maize acquired based on the point cloud color filtering algorithm, and (e) is the final point cloud model of maize acquired after down-sampling through the point cloud voxel filtering algorithm.

#### Euclidean clustering for image background removal

Euclidean clustering, as a clustering algorithm based on the Euclidean distance metric, essentially judges the distance between near-neighboring points by distinguishing the proximity of their neighborhoods. The KD-Tree based nearest neighbor query algorithm is one of the important preprocessing methods to accelerate the Euclidean clustering algorithm.

A point in the space was randomly selected as the initial point, and the Euclidean distance between each sample point and the initial point was calculated by the KD-Tree nearest neighbor search algorithm. If it is less than the Euclidean distance threshold, the point clouds are clustered into the most similar classes. Besides, the process is repeated until the number of point clouds no longer increases, and the whole clustering process is finished.

#### Point cloud filtering based on color threshold

In terms of the fundamental idea of color-based threshold segmentation in 2D images, it is to determine a threshold value, compare the grayscale value of each pixel with the threshold value, and classify the pixels according to the comparison result. The point cloud filtering based on color threshold is similar to this, and the RGB color threshold was determined after the RGB information of the point cloud was obtained.

Since there was observable difference between the RGB value of the point cloud noise and the RGB value of the leaf, the threshold was determined based on the difference. From the point cloud color information, the white noise at the edge of the corn seedling leaves was removed.

First, point cloud files were input to traverse all points in point clouds and acquire the RGB value of each point cloud. The values of each point cloud in three channels—R, G and B—were denoted as r, g and b, respectively, the sum of which was defined as *S*_rgb_. The absolute value of the difference value between r and g was solved as *abs*_rg_, that between b and g as *abs*_bg_, and that between r and b as *abs*_rb_, specifically as seen in Formula (1).


(1) $$\left\{ \begin{array}{c} S_{rgb}=r+g+b\\ \mathrm{ab}s_{rg}=|r-g|\\ \mathrm{ab}s_{bg}=|b-g|\\ \mathrm{ab}s_{rb}=|r-b| \end{array}\right.$$

The *abs*_rg_/*S*_rgb_ ratio was defined as *R*_rg_, the *abs*_bg_/*S*_rgb_ as *R*_bg_, and the *abs*_rb_/*S*_rgb_ ratio as *R*_rb_, as seen in Formula (2).


(2) $$\left\{ \begin{array}{c} R_{rg}=\mathrm{ab}s_{rg}/S_{rgb}\\ R_{bg}=\mathrm{ab}s_{bg}/S_{rgb}\\ R_{rb}=\mathrm{ab}s_{rb}/S_{rgb} \end{array}\right.$$

By inquiring RGB color values, the threshold distribution range of green color value is as seen in Table 1.

**Table 1:** Color threshold distribution of corn point cloud.

Color	Threshold range parameters	
	Min	Max
*S*_rgb_	165	642
*abs*_rg_	35	255
*abs*_bg_	16	255
*R*_rg_	0.098	0.697
*R*_bg_	0.032	0.670

When the point cloud part of maize met the above threshold distribution range, g > r and g > b, this point cloud was reserved as the maize plant part. If the above conditions were not satisfied, the point cloud was removed as a noisy point.

#### Point cloud down sampling based on voxel filtering

The purpose of point cloud voxel filtering is to reduce the number of point clouds using voxelization methods. Point cloud voxel filtering is the creation of tiny spatial 3D cubes, or voxel grids, in the point cloud data. All points in each voxel are approximated by its center of gravity, which enables point cloud down sampling. This method reduces the number of point clouds and keeps the morphological features of the point clouds unchanged. In addition, it is also useful in improving the speed of algorithms such as point cloud alignment and shape recognition. This method does not affect the microstructure of the point cloud compared to the random down sampling method, and the voxel-based filtering method is more accurate for the representation of surfaces corresponding to the sampled points.

#### Maize phenotype calculation method

The selection of a suitable calculation method is crucial to obtain accurate values of maize phenotypic parameters.

The corn plant height measured in this paper refers to the distance from the point where the plant meets the soil to the top of the corn seedling. Firstly, we use the translation and rotation matrix to align the growth direction of the maize seedlings with the positive direction of the z-axis, then we traverse all the point clouds to find the maximum and minimum values of the maize seedling point clouds on the z-axis, and finally we find the height of the maize by taking the difference.

The calculation formula is shown in Equation 3.


(3) $$h=z_{\text{max}}-z_{\text{min}}$$

The formula *h* indicates the height of the maize plant, *z*_max_ indicates the maximum value of the maize point cloud on the *z*-axis, and *z*_min_ indicates the minimum value of the maize point cloud on the *z*-axis.

In this paper, we use the RANSAC method of fitting a straight line to calculate the stalk height of maize. The Randomized Sampling Consensus (RANSAC) algorithm can estimate the parameters of a mathematical model from a set of observed data containing outliers using an iterative approach. The algorithm has a wide range of applications in linear fitting.

The algorithm is applied to the spatial straight line fitting with the following parameter settings. Firstly, *M* iterations are performed to find out the parametric model containing the maximum number of interior points, and then a subset of samples of size *n* is set to perform the calculation during the iteration. The point cloud model studied in this paper is a 3D model, so *n* = 2 is set and the iteration is stopped when *M* satisfies the following conditions.

The calculation formula is shown in Equation 4.


(4) $$M\geq \frac{\ln\left(1-P\right)}{\ln\left(1-1^{n}\right)}$$

where *P* is the degree of confidence, which is generally set to 99%.

After finding the fitted straight line of the maize stem, then calculate the point cloud coordinates of the two endpoints of the line based on the Euclidean distance calculation formula to find the stem height of the maize.

In this paper, maize seedlings are selected for the study, and the leaves at this stage are characterized by a small degree of curl. Therefore, the calculation of the leaf width and length of maize leaves was performed using an interactive point selection measurement method. The point cloud coordinates of the widest point in the transverse direction of the maize leaf and the longest point in the longitudinal direction of the maize leaf were manually selected.

Afterwards, the leaf width and leaf length are calculated based on the Euclidean distance algorithm formula.

Suppose the coordinates of two point clouds are p_1_ (*x*_1_, *y*_1_, *z*_1_) and p_2_ (*x*_2_, *y*_2_, *z*_2_), then the Euclidean distance calculation formula in 3D space is shown in Equation 5.


(5) $$d_{12}=\sqrt{{\left(x_{1}-x_{2}\right)}^{2}+{\left(y_{1}-y_{2}\right)}^{2}+{\left(z_{1}-z_{2}\right)}^{2}}$$

### Point cloud coordinate scale transformation

In order to obtain the dimensional relationship between the plant point cloud in 3D virtual space and the real-world plant, it is necessary to find the corresponding reference to calculate the scaling ratio. For acquiring the scale of the corn 3D point cloud model and the real corn plant, we used the checkerboard grid as the reference to calculate the conversion scale.

The calculation formula is Equation 6.


(6) $$k=\frac{L_{\mathrm{real}}}{L_{\mathrm{virtual}}}$$

Where *L*_real_ represents the real length of the checkerboard grid, *L*_virtual_ represents the length of the reconstructed model of the checkerboard grid, and k represents the conversion ratio. During the recording process, a checkerboard grid of a certain size (25 mm × 25 mm/grid) was placed by the plant. The actual size of the reconstructed maize model in the real world could be obtained after the calculation of the conversion ratio.

### Point cloud segmentation

In this paper, we used the region growth segmentation algorithm to segment the point clouds of maize seedlings. This method can better identify and segment plant organs such as the leaves and stems of maize seedlings. The principle of the region growth algorithm is to gather point clouds with similarities to form a region. Firstly, a seed point was identified for each region to be segmented as the starting point of the growth. Secondly, the points in the neighborhood around the seed point that had the same or similar properties to the seed were merged into the region where the seed pixel was located. Then the new points continued to grow like seeds in all directions until no more points satisfying the conditions could be included. In this algorithm, the output data structure is an array of clusters, where each cluster is a collection of points considered to be part of the same smooth surface. Moreover, the point clouds segmented using the area growth algorithms have one cluster for each color.

The region growth segmentation algorithm is mainly based on the specific implementation of normal difference and curvature difference. Firstly, the normal and curvature are calculated and sorted in ascending order according to the curvature. Secondly, the lowest curvature is selected as the initial seed point, and the neighboring points around the seed point are compared with the seed point. Finally, the normal angle threshold is set to determine whether the normal angle is smooth enough, and the curvature difference threshold is set to determine whether the curvature is small enough. If the normal angle threshold and curvature difference threshold are satisfied, the point can be used as the seed point. If only the normal angle threshold is satisfied, the point is classified without seeding.

## Results

### Point cloud reconstruction results

**Figure 3:**
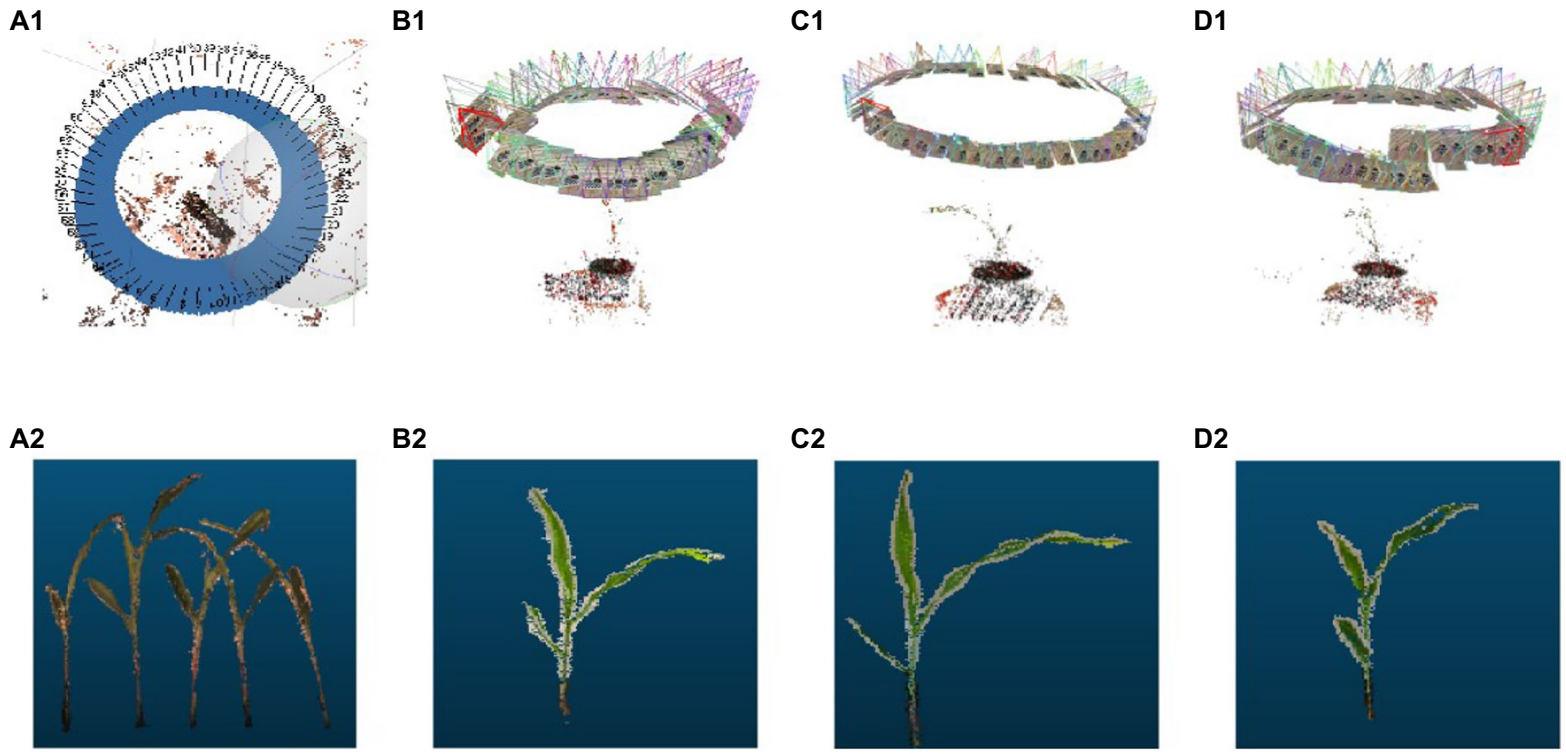
3D point cloud reconstruction process. **(A1)** The point cloud sparse reconstruction based on Agisoft Metashape. **(B1–D1)** 3D point cloud sparse reconstructions based on Visual SfM. **(A2–D2)** Point cloud extractions of maize seedlings.

After acquisition of the multi-view image sequence, the SfM algorithm was used to obtain the sparse point cloud. The Multi-View Stereo Reconstruction algorithm was then applied to reconstruct the sparse point cloud into a dense point cloud. Figure 3 shows the 3D point cloud reconstruction results of the image sequences extracted by these two methods.

### Point cloud pre-processing results

**Figure 4:**
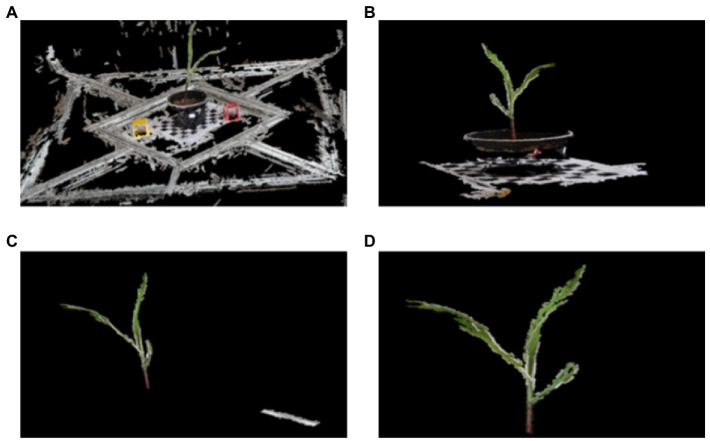
Segmentation Chart of Maize Seedlings Based on Euclidean Clustering. The segmentation process from **(A–D)** shows that the irrelevant background segmentation such as carrier table, flower pot and checkerboard grid can be removed using the Euclidean clustering segmentation algorithm.

Euclidean clustering algorithm was used to segment the plant point cloud and remove the irrelevant background and spatial discrete points. As shown in Figure 4, the algorithm could extract the 3D point cloud of corn seedlings in an intact way.

**Figure 5:**
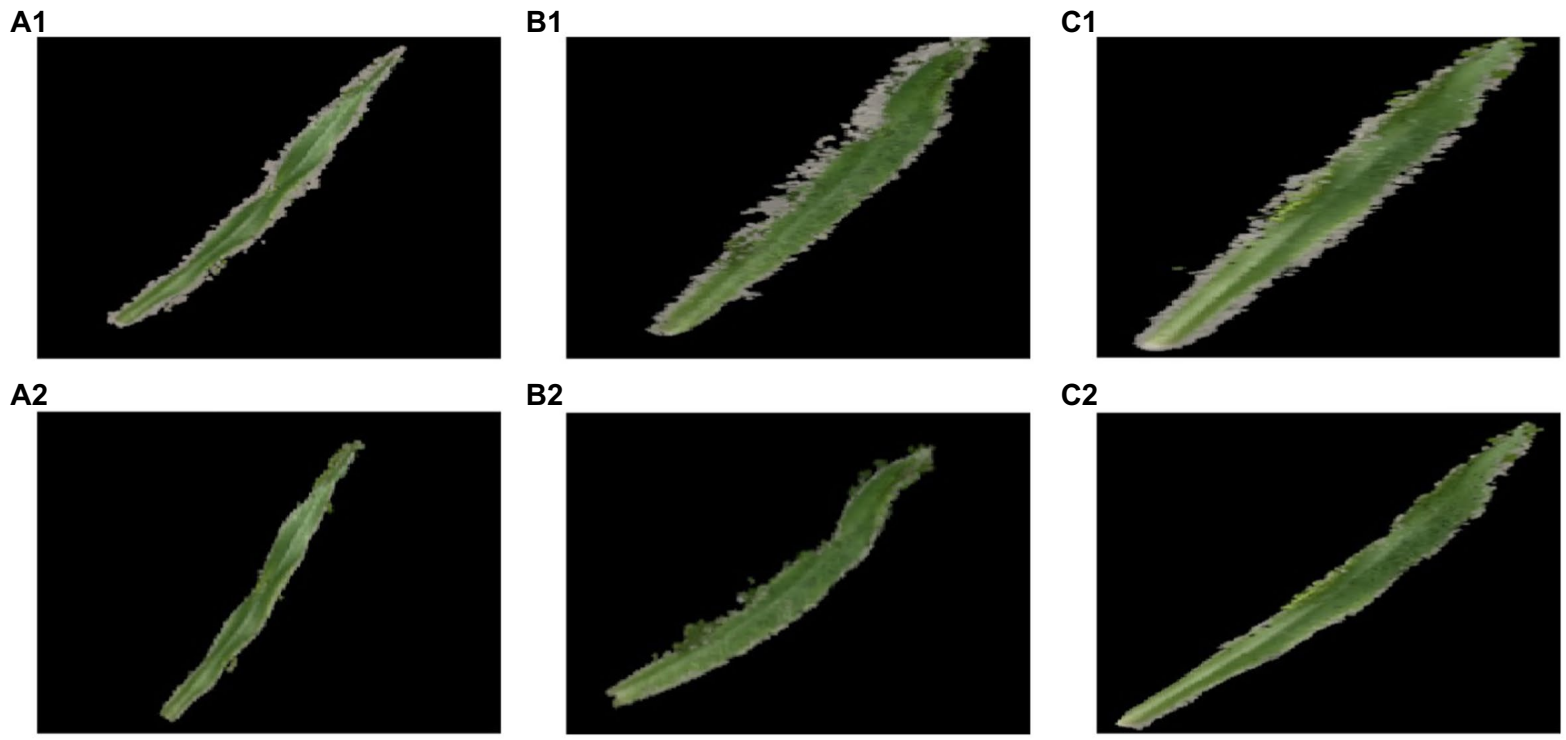
Color threshold based point cloud filtering of corn leaves. **(A1-C1)** Corn leaf point cloud images containing noise before filtering. **(A2-C2)** The corn leaf point clouds without noise after filtering.

As shown in Figure 5, a lot of point cloud noises were contained in the 3D point cloud images in (a1–c1) in addition to maize seedlings. As observed from the images in (a2–c2) after point cloud filtering, the white noisy point clouds at the leaf edge were obviously reduced, indicating the good point cloud filtering performance of this algorithm.

**Figure 6:**
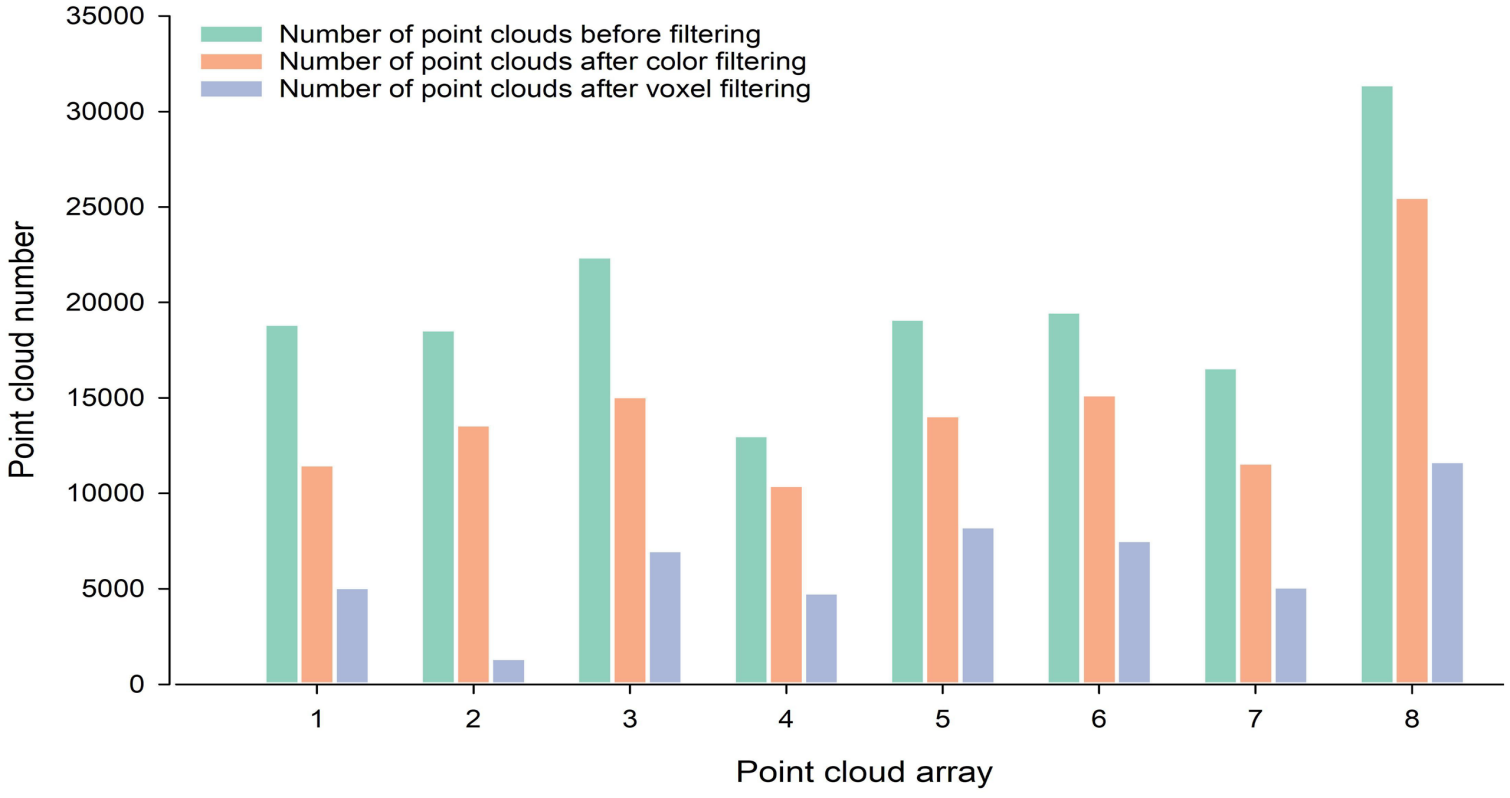
Number of point clouds before and after filtering. The three colors indicate the number of point clouds before filtering, the number of point clouds after color filtering, and the number of point clouds after voxel filtering, respectively.

The point cloud color filtering algorithm and the point cloud voxel filtering algorithm used in this paper had achieved good results in filtering the corn point clouds. Apart from that, eight groups of data were randomly selected from the acquired raw point cloud data for the point cloud filtering process. As shown in Figure 6, the number of point clouds in each group after filtering is significantly reduced compared with the number on point clouds before filtering.

### Maize point cloud segmentation recognition results

**Figure 7:**
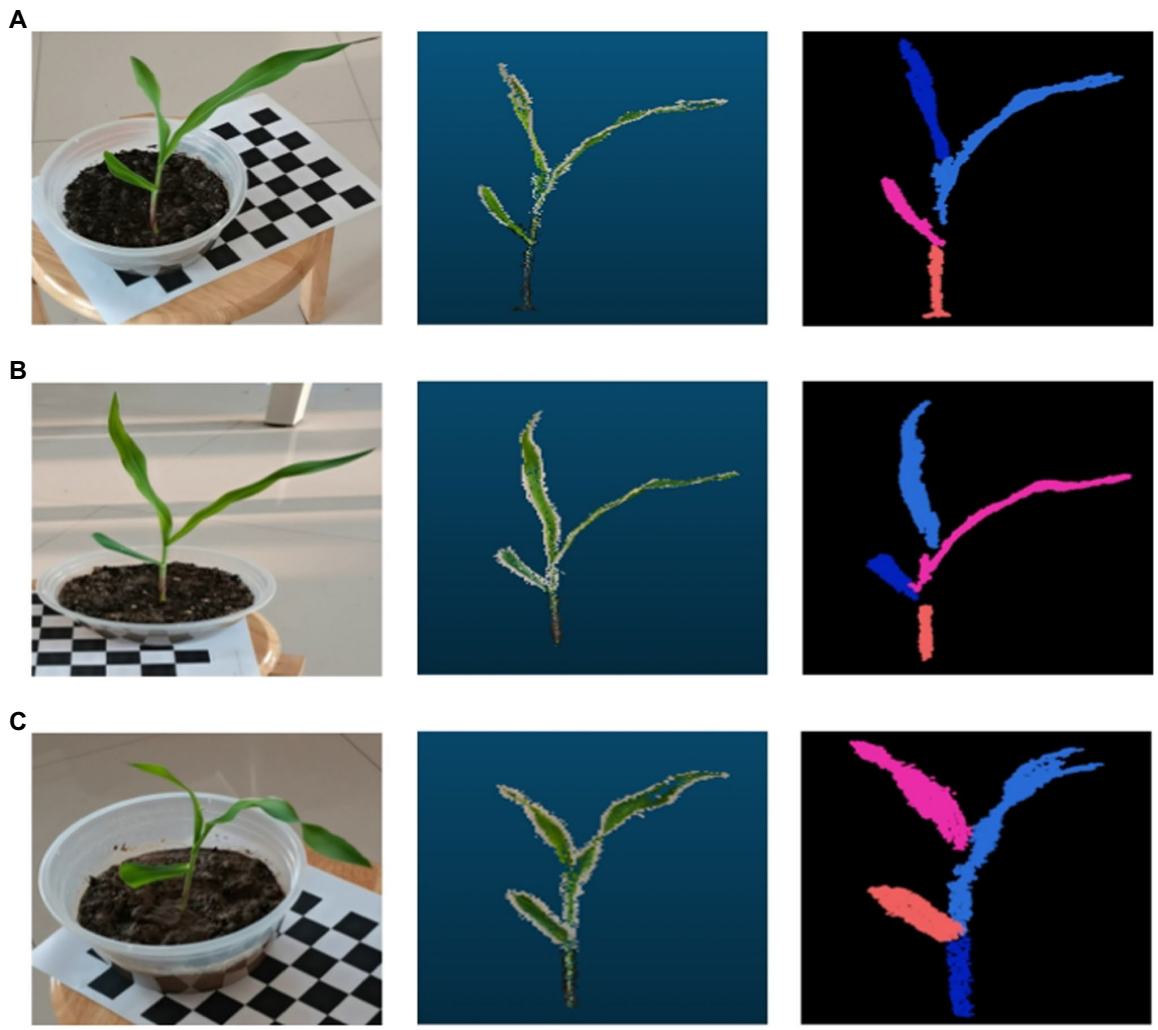
Maize point cloud segmentation based on region growth. In the three data sets **(A-C)**, the image on the left is when the data were collected, the middle one is the processed 3D point cloud image, and the image on the right is the corn point cloud after segmentation by the region growing algorithm.

The number of leaves of the maize plants all yielded similar results to the real plants when segmented using the region growth segmentation algorithm. As shown by the experimental result, the region growth algorithm used in this paper can not only segment and identify the number of leaves of maize more accurately, but also accurately identify the segmentation of its stems and leaves, and other organs (see the result of maize point cloud segmentation in Figure 7).

### 3D point cloud accuracy analysis of maize phenotype

**Figure 8:**
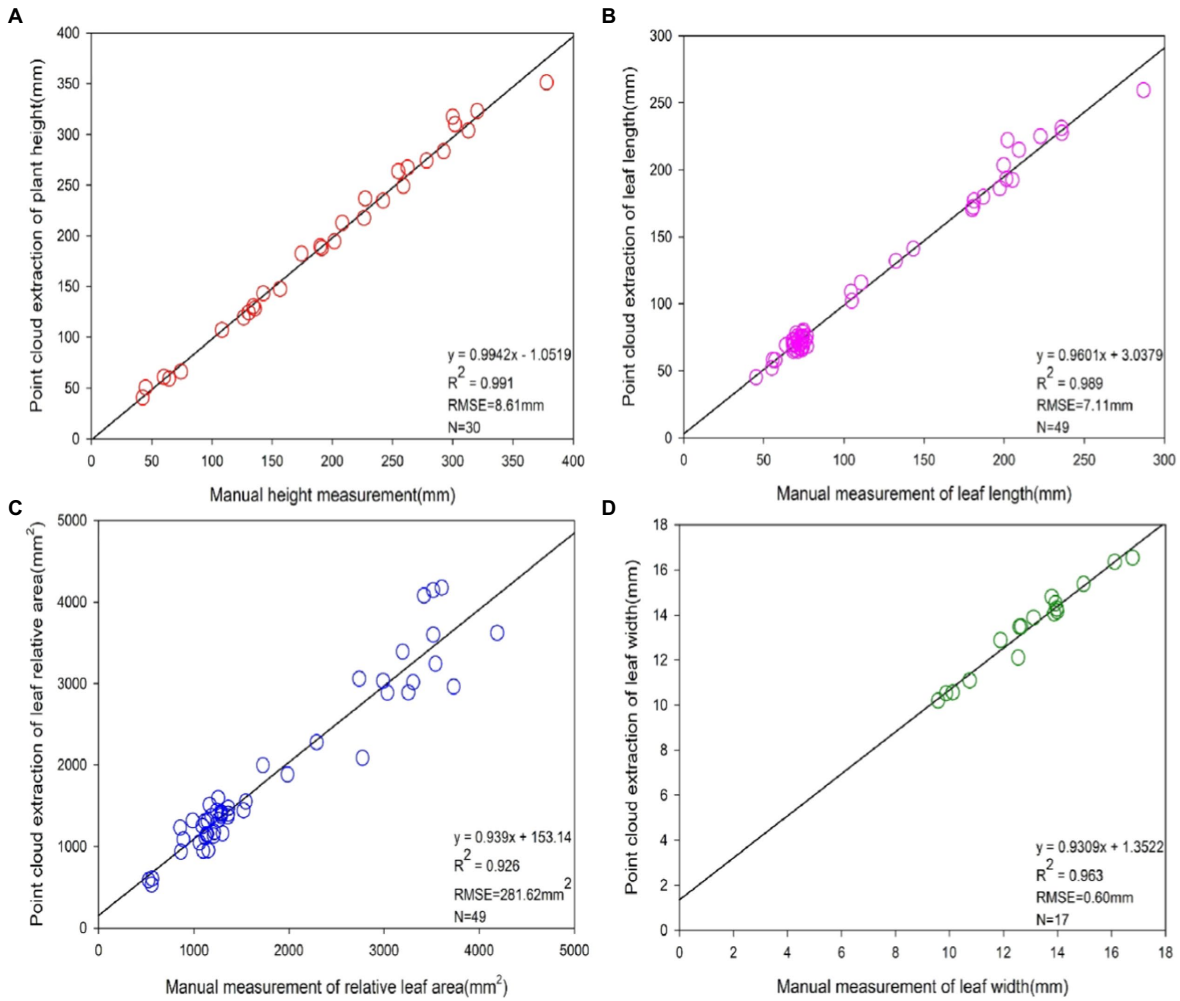
Accuracy analysis of phenotype extraction values based on 3D point cloud and manual measurements. From **(A–D)**, the precision analysis of phenotypes of plant height, leaf length, leaf relative area and leaf width based on 3D point clouds and manual measurements, respectively. The sample sizes N for four of the phenotypic indicators were 30, 49, 49 and 17, respectively.

A comparison of the point cloud extraction results of each phenotypic parameter of maize with the manual measurement results is displayed in Figure 8. Based on the obtained point cloud model, phenotypic parameters such as plant height, leaf length, relative leaf area, and leaf width of maize were calculated. Apart from that, 30 sets of plant height values, 49 sets of leaf length values, 49 sets of relative leaf area values and 17 sets of leaf width values were extracted by algorithmic measurements. The plant height, leaf length, relative leaf area values and leaf width values extracted through point cloud had a significant linear relationship with the manually measured values. The coefficients of determination *R*^2^ were 0.991, 0.989, 0.926 and 0.963, respectively. Besides, the root means square error RMSE was 8.61 mm, 7.11 mm, 281.62 mm^2^ and 0.60 mm, respectively.

Through the comparison between the point cloud measurements and the corresponding manual measurements, it was concluded that the algorithm used in this paper was accurate in maize reconstruction with multi-view imaging. This verifies, to a certain extent, that the method of extracting phenotypic parameters of crops *via* 3D imaging has high practicality and stability, and can extract the phenotypic parameter values of plants rapidly and losslessly.

### Error analysis

A total of 15 groups of measured maize data were selected to analytically evaluate their errors as seen in Table 2, where *L*_0_, *W*_0_, *S*_0_ and *H*_0_ denote the actual leaf length, leaf width, relative leaf area and maize plant height acquired through artificial detection, respectively, and *L*, *W*, *S* and *H* stand for those measured through point cloud computing, respectively.

**Table 2:** Measured data of phenotypic parameters of maize.

Corn Number	*L*_0_ (mm)	*L* (mm)	*W*_0_ (mm)	*W* (mm)	*S*_0_ (mm^2^)	*S* (mm^2^)	*H*_0_ (mm)	*H* (mm)
1	64.00	69.17	13.97	14.29	1113.60	1308.14	74.20	66.15
2	69.20	66.06	13.88	14.07	1141.80	1155.54	44.80	50.55
3	45.20	45.14	16.12	16.36	863.32	935.93	42.40	40.52
4	70.70	71.63	14.97	15.38	855.47	1230.96	64.40	59.10
5	70.10	77.67	13.11	13.88	1184.69	1371.95	60.00	60.86
6	104.40	108.94	16.78	16.54	1545.12	1551.94	135.70	128.24
7	104.90	102.05	10.75	11.09	1521.05	1441.25	126.30	119.19
8	71.20	64.87	12.54	12.11	1132.08	1147.46	108.20	107.02
9	132.40	131.95	12.66	13.48	1986.00	1881.59	134.50	130.37
10	110.70	115.75	13.99	14.16	1726.92	1994.75	130.40	124.37
11	180.10	170.69	12.59	13.48	2773.54	2084.64	174.50	182.54
12	180.70	172.21	13.79	14.80	3035.76	2887.03	156.70	147.38
13	143.30	141.21	10.13	10.56	2292.80	2278.85	142.70	143.12
14	197.30	186.36	9.59	10.20	3255.45	2890.01	201.90	194.59
15	181.20	177.19	9.88	10.52	2989.80	3033.35	191.20	187.77

Two indexes—absolute error and relative error—were used to evaluate and analyze the errors generated by point clouds, where the former means the absolute difference value between measured value and real value, and the latter stands for the percentage of absolute error in the real value. In this study, the manually measured phenotypic parameter values of maize served as real values, and those obtained through point cloud computing as measured values. The absolute error and relative error of such phenotypic parameters and their average errors are listed in Table 3.

**Table 3:** Error analysis of phenotypic parameters of maize.

Corn number	Leaf length		Leaf width		Relative leaf area		Plant height	
	Absolute error (mm)	Relative error (%)	Absolute error (mm)	Relative error (%)	Absolute error (mm^2^)	Relative error (%)	Absolute error (mm)	Relative error (%)
1	5.17	8.08	0.32	2.29	194.54	17.47	8.05	10.85
2	3.14	4.54	0.19	1.37	13.74	1.20	5.75	12.83
3	0.06	0.13	0.24	1.49	72.61	8.41	1.88	4.43
4	0.93	1.32	0.41	2.74	375.49	43.89	5.3	8.23
5	7.57	10.80	0.77	5.87	187.26	15.81	0.86	1.43
6	4.54	4.35	0.24	1.43	6.82	0.44	7.46	5.50
7	2.85	2.72	0.34	3.16	79.8	5.25	7.11	5.63
8	6.33	8.89	0.43	3.43	15.38	1.36	1.18	1.09
9	0.45	0.34	0.82	6.48	104.41	5.26	4.13	3.07
10	5.05	4.56	0.17	1.22	267.83	15.51	6.03	4.62
11	9.41	5.22	0.89	7.07	688.9	24.84	8.04	4.61
12	8.49	4.70	1.01	7.32	148.73	4.90	9.32	5.95
13	2.09	1.46	0.43	4.24	13.95	0.61	0.42	0.29
14	10.94	5.54	0.61	6.36	365.44	11.23	7.31	3.62
15	4.01	2.21	0.64	6.48	43.55	1.46	3.43	1.79
Average error	4.74	4.32	0.50	4.06	171.90	10.51	5.08	4.93

Table 2 displays the errors between manually measured values and point cloud computed values of maize seedlings. It could be seen from average absolute errors that the absolute errors of maize leaf length, relative leaf area and plant height were all large. The main reason was that the leaf length was mainly measured the straight line distance between the two ends of the leaf veins of the leaves, and did not fully consider the degree of curvature of the corn leaves. Moreover, the relative leaf area was approximately expressed by the product between leaf length and leaf width, and its absolute error was greatly influenced by the leaf length. The measurement of maize plant height was affected by soil factors, which disturbed the accurate measurement to some extent. The error in the measurement of maize plant height comes from the following two main components. In this paper, the stalk portion buried by soil is not included in the measurement of maize plant height. This part of the error exists for both manually measured and point cloud extracted maize plant height. Therefore, the maize plant height defined in this paper is the distance from the contact of the maize with the soil part to the highest point of the top of the maize. In addition, the point cloud extracted maize plants have a small portion of soil on the stalk near the soil part. This part is sometimes removed when performing plant height calculations. However, during the manual measurement, this part of the stalk with soil can be measured accurately. Therefore, there is some error in the point cloud extracted plant height compared to the actual measurement.

According to the average relative errors of maize phenotypes, the relative errors of maize leaf length, leaf width and plant height were relatively approximate, while the average relative error of relative maize leaf area was relatively large, which might be ascribed to the not intact enough local point clouds during the 3D maize reconstruction. Hence, the deviation of a minority of calculated relative leaf area data was large.

### Results on the growth dynamics of maize seedlings based on 3D model

**Figure 9:**
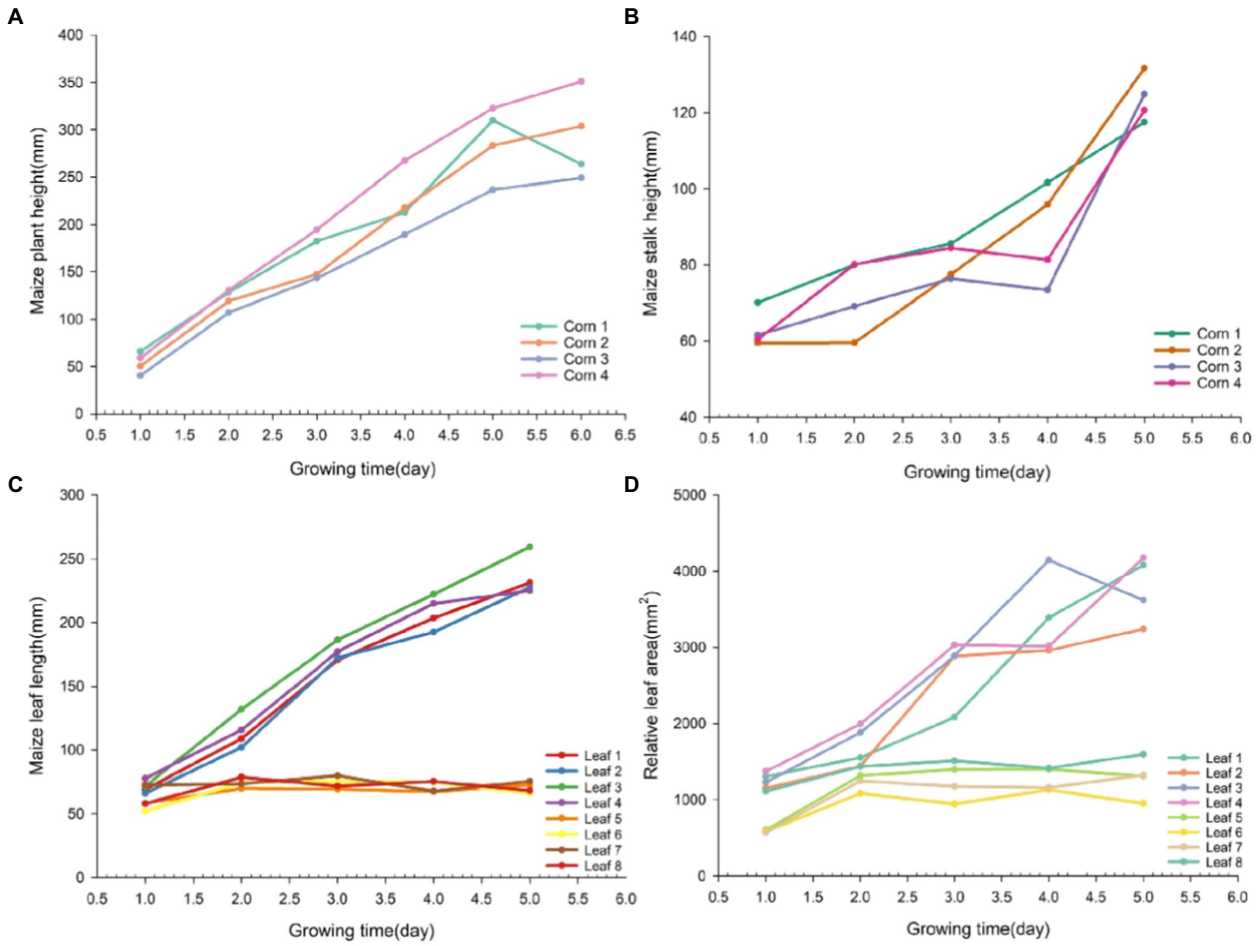
Line graph of growth dynamics of different phenotypic traits in maize. Maize phenotypic traits were plant height **(A)**, stem height **(B)**, leaf length **(C)**, and relative leaf area **(D)** in that order.

Four plants were randomly selected for growth tracking study, with the longest leaves from each plant numbered as Leaf 1–4 and the one of the shorter leaves numbered as Leaf 5–8. Figure 9 shows the dynamics of phenotypic parameters such as plant height, stem height, leaf length and relative leaf area of seedling numbered maize plants over 1 week. In the early stages of growth, all phenotypes presented an increasing trend. Among the four traits, the growth rate of plant height and stem height varied more significantly. Beyond that, the dynamics of leaf length and relative leaf area varied more significantly from leaf 1 to 4 and less significantly from leaf 5 to 8. By observing the changes in the phenotypic parameters of maize in Figure 9, we can find out that the growth rate of maize seedlings varies among different individuals. The differences in the growth rate of maize can reveal to some extent how well it is growing. It can be seen that the analysis of phenotypic parameters of maize can provide unique insights for its development in precision agriculture.

## Discussion

With the continuous development of 3D reconstruction technology, the research of the 3D reconstruction of crops based on various sensors has also made significant progress (Gibbs et al., 2017). The traditional phenotypic analysis methods of plants are characterized by destructiveness, great time consumption, low efficiency and high cost, so the present research focus has been on nondestructively, rapidly, and accurately acquiring plant phenotypes. The most extensively applied 3D reconstruction technologies for plant phenotypes mainly include image technology-based 3D reconstruction, laser radar technology-based 3D reconstruction and 3D reconstruction based on UAVs in combination with various sensors (e.g., multispectral cameras, hyperspectral sensors or lidars). When applied to the analytical investigation on plant phenotypes, UAVs integrate the merits of portability, high efficiency and suitability for field operation. However, UAV detection is also influenced by other limiting factors, such as weather effect, limited working altitude in the air, and limited data processing speed (Zhao et al., 2019). Despite the capability of 3D imaging, multispectral and hyperspectral cameras are sensitive to electromagnetic radiation spectra with a broad scope, and fail to detect the specific information from a single wavelength (Tripodi et al., 2018). In addition, most spectral cameras cannot be promoted in a large scale due to the high cost. The development lidars has provided an effective analysis tool for investigating indoor and field plant phenotypes and improved the 3D plant modeling at different spatial–temporal scales in agriculture. The plant phenotypes extracted by lidars play important roles in agricultural seed breeding and management by virtue of a very high accuracy (Jin et al., 2021). However, the difficulty of lidars lies in how to realize high-speed data acquisition through hardware and their real-time processing using algorithms so as to acquire high-accuracy original point cloud data. In this study, the image-based 3D reconstruction method with simple operations was used, where the RGB camera, which was cheap, could be integrated onto the self-established phenotype platform, thereby providing an effective solution to the extraction of crop phenotypes. In addition, studies on 3D reconstruction of maize have been more focused on the ears and grains stage (Ma et al., 2019; Wang et al., 2019; Zhu et al., 2020; Zhang et al., 2022a), while less attention has been paid to 3D reconstruction of seedlings. During the ears and grains stage, the maize structure is complex and it is more difficult to perform 3D reconstruction of maize. In contrast, at the seedling stage, the maize plant has few leaves and simple growth structures, so 3D reconstruction of maize at the seedling stage is easier to accomplish. In this paper, we have successfully achieved 3D reconstruction and phenotype extraction of maize seedlings.

Changes in phenotypic parameters during the growth of maize seedlings are important observations reflecting their growth. The size of leaves, plant height and stem height of maize seedlings are important phenotypic information that represent their growth rate and seed vigor (Das Choudhury et al., 2020). Therefore, we designed an image acquisition platform for the 3D reconstruction of maize seedlings after acquiring multi-view image sequences, and calculated the actual size of the maize phenotype with a checkerboard grid.

Compared to 3D reconstruction with devices such as LiDAR and Kinect, the method we used only requires the use of RGB cameras and video frame extraction to acquire image sequences, which is more convenient for acquiring data, faster and more automated when performing 3D reconstruction (Garrido et al., 2015; Sun and Wang, 2019). Thapa et al. (2018) measured the total leaf area of maize and sorghum using a lidar, with *R*^2^ values of 0.95 and 0.99, respectively, indicating a high measurement accuracy. In this study, the mean *R*^2^ value of image-based maize phenotype measurement could also reach 0.967, which differed very little from the model accuracy in geometrical measurement after lidar-based 3D reconstruction. Besides, the image-based method could acquire more detailed phenotype information in comparison with the lidar-based 3D reconstruction method. The multi-view image-based 3D reconstruction could not only observe the morphological characteristic information plants but also observe their color characteristic information. The color information could represent the plant growth status, based on which countermeasures could be taken in advance to ensure the healthy growth of crops.

In terms of the point cloud segmentation, the region growth algorithm used could achieve more accurate stalk segmentation recognition of corn seedlings. However, in order to obtain the optimized segmentation results, it was required to adjust the threshold values for point cloud segmentation step by step, which was a time consuming process. In recent years, researchers have investigated the skeletonization of crop point cloud models (Wu et al., 2019; Liu et al., 2021a), which provides a new research idea for point cloud segmentation. In conclusion, the effective segmentation of point clouds still needs more exploration and further research.

Compared with 2D imaging, 3D reconstruction can obtain more detailed morphological characteristics of crop phenotypes, but it could time consuming depending on the number of reconstructed point clouds and hardware equipment. In our study, it usually took 30 ~ 40 min to perform a set of 3D model reconstruction. Therefore, it is necessary to consider reducing the quality of the point cloud reconstruction as well as the number of point clouds without affecting the reconstruction effect, so as to accelerate the point cloud reconstruction process. The extraction of phenotypic parameters after 3D reconstruction of maize was influenced, to some extent, by such objective factors as incomplete experimental methods and equipment and the surrounding environment, and measurement errors were thus generated. Specifically, such errors mainly derived from the minor vibration of the rotating platform of the 3D imaging device during the rotation as well as out-of-focus situation during the photographing of RGB camera due to inadequate lighting. Consequently, a minority of acquired pictures were unclear, thus generating point cloud noises. The indoor environment was complicated, the 3D imaging device was not isolated using a background plate, so unrelated backgrounds during the photographing process were also recorded, thus leading to point clouds of unrelated backgrounds generated in the 3D reconstruction of maize.

## Conclusion

In this study, the phenotypes of maize seedlings were investigated using the multi-view image-based 3D reconstruction method, including the following three parts.

In the first stage, the self-designed 3D imaging device was used to acquire image data, and a multi-view image sequence was acquired through an RGB camera and video frame extraction method, followed by the 3D reconstruction of maize seedlings based on SfM algorithm. Subsequently, the acquired original point cloud data of maize were preprocessed using the Euclidean clustering algorithm, point cloud color filtering algorithm and point cloud voxel filtering algorithm, thus obtaining a point cloud model of maize. In the second stage, the images acquired by the RGB camera were used for the 3D reconstruction of maize, and then the phenotypic parameters of maize obtained by point cloud computing were compared with the corresponding manually measured values. The mean *R*^2^ value could reach 0.967. In addition, the point cloud errors of maize phenotypes were analyzed, which tended to be small, indicating a high accuracy of the established 3D reconstruction model of maize in this study. Therefore, the proposed method was applicable to the phenotypic analysis of maize crops and feasible in analyzing maize phenotypes. In the third stage, the 3D reconstruction of maize was performed through the video frame extraction method, which was featured by a high speed and less time consumption. Afterwards, maize stem leaves were segmented and identified through the region growing segmentation algorithm, and the expected segment effect was harvested.

To sum up, the proposed 3D reconstruction method, which is characterized by automation, high efficiency and nondestructive extraction in the research on corn phenotypes, can provide guidance for maize breeding and growth monitoring.

## Data availability statement

The raw data supporting the conclusions of this article will be made available by the authors, without undue reservation.

## Author contributions

YL: writing-original draft. JL: guiding and supervision. XZ and BF: data collection. XL and YH: data process. BZ, JY, and YW: participating in the discussion. XF: editing, supervision, and proofreading. All authors contributed to the article and approved the submitted version.

## Funding

This study was supported by the National Natural Science Foundation of China (32072572), Hebei Talent Support Foundation (E2019100006), Key Research and Development Program of Hebei Province (20327403D), the Talent Recruiting Program of Hebei Agricultural University (YJ201847), Fundamental Research Funds Project of Hebei Agricultural University (KY2021023), and the University Science and Technology Research project of Hebei project (QN2020444). Detection of crop diseases and insect pests based on artificial intelligence and multispectral imaging (2021 Shijiazhuang City, the introduction of foreign technology projects).

## Conflict of interest

YW was employed by the company Hebei Runtian Water-saving Equipment Co., Ltd.

The remaining authors declare that the research was conducted in the absence of any commercial or financial relationships that could be construed as a potential conflict of interest.

## Publisher’s note

All claims expressed in this article are solely those of the authors and do not necessarily represent those of their affiliated organizations, or those of the publisher, the editors and the reviewers. Any product that may be evaluated in this article, or claim that may be made by its manufacturer, is not guaranteed or endorsed by the publisher.
